# A New and Original Method of Treating Exposed Pulps

**Published:** 1882-01

**Authors:** William Victor Ditcham


### A NEW AND ORIGINAL METHOD OF TREATING EXPOSED PULPS.

Every educated member of the profession will agree with me when I say that the preservation of exposed pulps is a most important operation, and one the results of which, up to the present, are far from what they might be. I have lately been experimenting upon an entirely original method for their preservation, with very gratifying results. The procedure is as follows :

Procure a piece of clean metal (that used for working celluloid will do very well), about No. 6 in thickness, and cut a square piece the size of the bottom of the cavity to be operated in ; in the center of this cut a small hole large enough to receive a platinum tube (one taken from a tube tooth answers admirably) ; insert the tube in the center, and soft-solder together : the cap may be bent slightly convex. This is then placed at the bottom of the cavity, directly over the exposed pulp, resting on the dentine, and is fixed there by "fossiline," and completely covered with the same (I am here speaking of the cap only) —and, as far as oxychlorides go, up to the present I consider fossiline the best. This effectually holds the cap in position, and when once set, it is difficult to move. We can then fill with whatever material we consider suitable. If gold, a piece of pin wire, with a round head, kept for the purpose, should be inserted in the tube to protect it from being compressed during consolidation. We have thus a very direct and certain means of treating the pulp. Apply a small crystal of carbolic acid through the tube, and then close it by inserting a small screw with a rounded head. It is understood that a thread must be cut in the tube about one-sixteenth of an inch in length to effectually secure the screw, and thus completely seal the tube. The tube must be carefully adjusted evenly with the masticating surface, and with regard to the articulation of the antagonizing teeth. For the treatment of alveolar abscess, suppuration, etc., I believe it bids fair to become a very successful operation. We can thus treat the tooth mechanically at once, and leave the surgical treatment to be entirely independent. The screw can be removed by patients if necessary. We can treat a pulp with much less difficulty and trouble, and the annoyance of having to remove and insert fresh dressings, and seal up with mastic, etc., is entirely avoided. Further, we are saved the uncertainty of any pressure resulting from a freshly-inserted dressing, and thus irritating the pulp. I have performed this operation seventeen times, and in every case with success, and can assuredly say that the beneficial results were brought about in a much shorter time than by the previous method of dressings on wool, etc. When the treatment is complete, the screw is removed and substituted by gold foil or hickory wood (the latter I have not yet tried). I shall be glad if some members of the profession (careful operators) will test this operation and make known their experiences.—*William Victor Ditcham, in the Dental Record.*

